# Differential systemic exposure to galangin after oral and intravenous administration to rats

**DOI:** 10.1186/s13065-015-0092-5

**Published:** 2015-03-31

**Authors:** Feng Chen, Yin-Feng Tan, Hai-Long Li, Zhen-Miao Qin, Hong-Die Cai, Wei-Yong Lai, Xiao-Po Zhang, Yong-Hui Li, Wei-Wei Guan, You-Bin Li, Jun-Qing Zhang

**Affiliations:** Hainan Provincial Key Laboratory of R&D of Tropical Herbs, School of Pharmacy, Hainan Medical University, Haikou, 571199 China; Nanjing University of Chinese Medicine, Nanjing, 210046 China

**Keywords:** Galangin, Glucuronidation, Sulfation, Administration routes, LC-MS/MS

## Abstract

**Background:**

Galangin (3,5,7-trihydroxyflavone) is present in high concentrations in herbal medicine such as *Alpinia officinarum* Hance. Galangin shows multifaceted *in vitro* and *in vivo* biological activities. The number and position of hydroxyl groups in this molecule play an important role in these biological activities. However, these hydroxyl groups undergo glucuronidation and sulfation in *in vitro* assay system. However, the systemic exposure to galangin after dosing in animals and/or humans remains largely unknown. Thus it is not clear whether the galangin exists in the body at concentrations high enough for the biological effects. Furthermore, the metabolite identification and the corresponding plasma pharmacokinetics need to be characterized.

**Results:**

Two LC-MS/MS methods were developed and validated and successfully applied to analyze the parent drug molecules and aglycones liberated from plasma samples via β-glucuronidase hydrolysis. Our major findings were as follows: (1) The routes of administration showed significant influences on the systemic exposure of galangin and its metabolites. (2) Galangin was preferentially glucuronidated after p.o. dosing but sulfated after i.v. medication. (3) Kaempferol conjugates were detected demonstrating that oxidation reaction occurred; however, both glucuronidation and sulfation were more efficient. (4) Oral bioavailability of free parent galangin was very low.

**Conclusions:**

Systemic exposure to galangin and its metabolites was different in rat plasma between oral and intravenous administration. Further research is needed to characterize the structures of galangin conjugates and to evaluate the biological activities of these metabolites.

**Graphical abstract:**
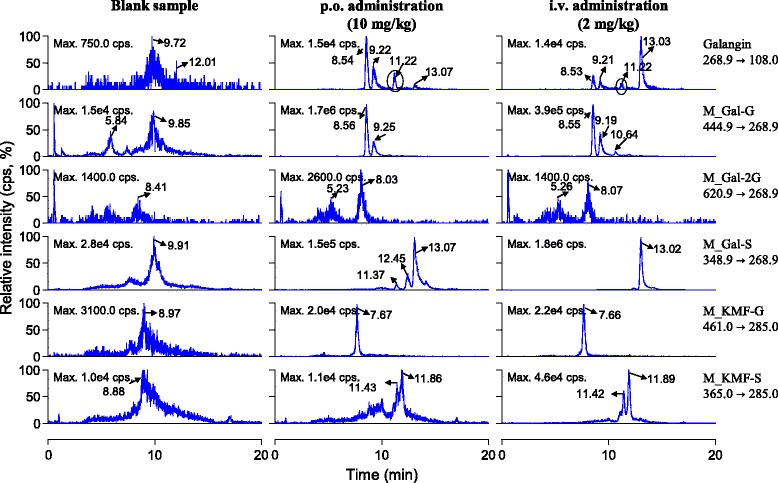
Galangin was preferentially glucuronidated after p.o. dosing but sulfated after i.v. medication.

## Background

Flavonoids constitute the most ubiquitous polyphenolic compounds in the human dietary sources (*e.g.* fruits, vegetables, tea and wine) and herbal medicines. Epidemiological studies suggest an inverse association between flavonoids intake and risks for certain disease such as cardiovascular and neurodegenerative disease and certain human cancers [1-3]. These beneficial roles are closely associated with their antioxidative activities, cardiovascular protection, neuroprotection, anti-inflammation and/or antitumor effects. Obviously, a majority of pharmacological assessments have been performed on the flavonoid aglycones (*e.g.*, quercetin, kaemfperol) [4]. However, flavonoids, once ingested, undergo extensive presystemic metabolism by methylation, glucuronidation and sulfation in the intestine and in the liver, resulting in very low concentration localizing in the body in its original form [5]. Therefore, accurate and complete pharmacokinetic (PK) information of flavonoid is useful for the pharmacological activity evaluation of unmetabolized chemicals and/or phase II conjugates substantially circulating in the bloodstream after dosing, which may enhance understanding of the final chemical entities that reach the target sites.

**Figure 1:**
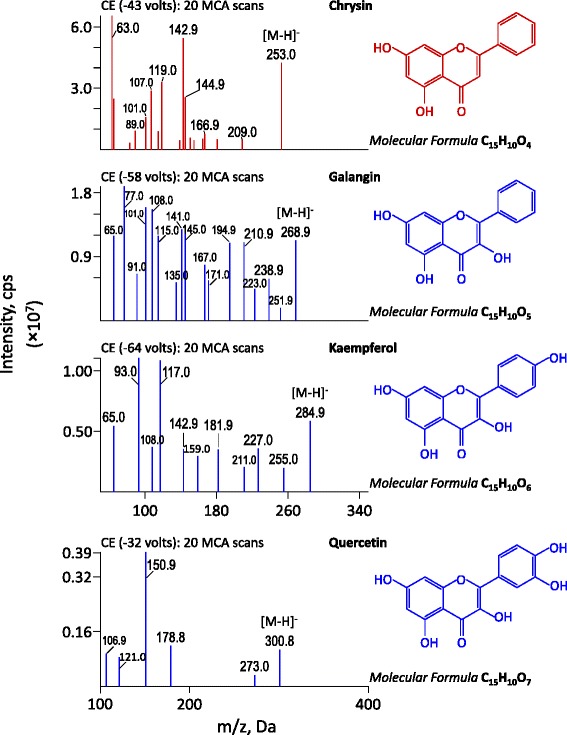
**Chemical structures and MS/MS spectra of galangin, kaempferol, quercetin and chrysin (IS, top panel).**

Galangin (3,5,7-trihydroxyflavone, Figure 1) is one of the main active component of *Alpinia officinarum* Hance. This herb has been used for counteracting the abdominal pain, emesis, diarrhea, impaired renal function and dysentery [6]. Galangin shows multifaceted biological activities. Galangin (20–80 μM) suppressed β-catenin response transcription (CRT), down-regulated the intracellular β-catenin levels, repressed β-catenin/T-cell factor-dependent genes expression and thus inhibited the CRT-positive cancer cells proliferation. Moreover, hydroxyl groups at positions 3-, 5- and 7- were critical for galangin-mediated β-catenin degradation [7]. Galangin (≥10 μM) significantly inhibited TNFα-induced adhesion molecule expression in human aortic endothelial cells and the A-ring 5, 7-dihydroxyl groups *per se* are required [8]. Galangin (50 μM) suppressed iNOS mRNA expression and COX-2 transcription during the inflammatory response [9]. The number and position of hydroxyl groups in galangin molecule involved in interacting to the alkyl chains of the lipid bilayer play an important role in membrane binding [10]. However, these functional groups underwent predominant glucuronidation and sulfation in freshly plated human hepatocytes [11]. Therefore, *in vitro* cell studies using relatively high concentrations of parent galangin cannot be directly extrapolated to the *in vivo* situation.

Oral administration of galangin (20 mg/kg) efficiently counteracted the anomalies induced by benzo(a)pyrene in male Swiss albino mice via the increased activity of phase I drug metabolic enzymes, lipid peroxidation levels, tissue marker enzymes and the decreased activity of phase II metabolic enzymes, antioxidant levels, as well as severe alveolar and bronchiolar damages and restored cellular homeostasis [12]. Intraperitoneal injection of galangin (5 and 15 mg/kg) to BALB/c mice dose-dependently inhibited ovalbumin-induced increases in total cell counts, eosinophil counts and IL-4, IL-5, IL-13 levels in bronchoalveolar lavage fluid and reduced the ovalbumin-specific IgE in serum. Galangin blocked κB degradation, phosphorylation of the p65 subunit of NF-κB and p65 nuclear translocation from lung tissues of ovalbumin-sensitized mice [13]. Overall, *in vivo* pharmacological studies have also confirmed that galangin has a plethora of beneficial biological effects. However, the systemic exposure to galangin after dosing in animals and/or humans remains largely unknown. Since galangin undergoes extensive phase II metabolism, it is not clear which species among galangin and its metabolites is mainly responsible for the observed in vivo pharmacological effects. To answer this question, it is necessary to characterize the pharmacokinetic behaviors of galangin and its metabolites.

The primary objective of this study was to identify galangin metabolites in rat plasma after oral (p.o.) and intravenous (i.v.) administration of galangin solution. Also, we investigated the pharmacokinetic behaviors of galangin and its metabolites after different routes of administration.

### Experimental

#### Chemicals and materials

Analytical reference standards of galangin, kaempherol and quercetin (Figure 1) were obtained from the National Institutes for Food and Drug Control (Beijing, China). Chrysin (used as internal standard, IS; Figure 1) was separated from *Alpinia oxyphylla* and identified in our lab. For galangin, kaempherol, quercetin, and chrysin, the purity is over 98.0%. L-Ascorbic acid (Vitamin C) was obtained from Biosharp Co. (Hefei, China). β-Glucuronidase (Type HP-2, from *Helix pomotia*, containing ≥ 100,000 unit/mL of β-glucuronidase and 7,500 unit/mL of sulfatase) was purchased from Sigma-Aldrich (St. Louis, MO, USA). Methanol and acetonitrile for HPLC use were products of Tedia Company Inc. (Fairfield, OH, USA). HPLC-grade formic acid (HCOOH) was purchased from Aladdin Industrial Inc. (Shanghai, China). Purified water was prepared in house using the Milipore system (Millipore, Bedford, MA, USA). The other chemical reagents of analytical grade or better were obtained from Hainan YiGao Instrument Co., Ltd (Haikou, China).

#### Experimental animals

The use of rats and study protocols were approved by the Institutional Animal Care and Use Committee at the Hainan Medical University (Haikou, China). Female Sprague-Dawley (SD) rats (204–248 g) were purchased from DongChuang Laboratory Animal Service Department (Changsha, China). Commercial rat chow was available *ad libitum* except for an overnight fasting period before dosing. All rats had free access to water.

#### Drug administration and blood collection

For the p.o. and i.v. administration, galangin solution (1 mg/mL) was dissolved in a mixture containing 6% (v/v) PEG400, 9.8% (w/v) Tween-80 and 4.4% (v/v) ethanol. The rats were randomly divided into two groups (3 rats/group) to receive oral (10 mg/kg) administration and intravenous (2 mg/kg) administration of galangin, respectively. Serial blood samples (0.3 mL; 0, 5, 15, 30 min and 1, 2, 4, 6, 8, 10 and 24 h post dosing) were collected into heparinized tubes. The blood samples were then centrifuged at 13,000 rpm for 10 min and the plasma fractions were decanted and frozen at –70°C until analysis.

#### Plasma sample clean-up

The rat plasma samples were treated via different methods for qualitative and quantitative analysis, respectively. The details were as follows:

#### Protein precipitation

For identification of galangin and its metabolites (qualitative analysis), 20 μL of each plasma sample collected at different time points (5, 15, 30 min and 1, 2 h) in the same group were pooled together. The resultant mixture (100 μL) was treated via vortex-shaking for 10 min with 300 μL methanol and then centrifuged at 13,000 rpm for 10 min. The upper supernatant (~390 μL) was dried under a stream of N_2_ via a Techne™ Sample Concentrator (Bibby Scientific Ltd., Staffordshire, UK). The residue was reconstituted in 50 μL of methanol, centrifuged *ditto*, and 10 μL of the resulting supernatant was applied for LC-MS/MS analysis.

For the quantification of free galangin, as well as aglycones liberated from its glucuronidated metabolites, aliquot (50 μL) of each plasma sample was precipitated with 150 μL of the IS-spiked acetonitrile solution (500 ng/mL). The mixture was vortex-shaked for 5 min and centrifuged at 13,000 rpm for 10 min. The resulting supernatant (10 μL) was directly applied for LC–MS/MS analysis.

#### Enzymatic hydrolysis coupled with protein precipitation [14]

Aliquots (50 μL) were treated with 150 μL of β-glucuronidase (2,000 unit/mL in pH 5 acetate buffer). 10 μL of ascorbic acid (1 mg/mL) was added and incubated at 37°C for 60 min. After incubation, the above plasma sample was mixed with 150 μL of acetonitrile containing the IS (500 ng/mL) and then vortex-extracted for 5 min and centrifuged at 18,140 *g* for 10 min. Ten microliters of the resulting supernatant were directly injected into LC–MS/MS system for analysis.

#### LC-MS/MS equipment

The LC-MS/MS system consisted of an AB-SCIEX API 4000 plus triple quadrupole mass spectrometer (Toronto, Canada) interfaced via ionization probe with a Shimadzu Prominence ultra fast liquid chromatography chromatographic system (Kyoto, Japan) including two pumps, a degasser unit, an auto-sampler and a column oven. The AB-SCIEX Analyst software packages were used for controlling the LC-MS/MS system, data acquisition and processing.

#### Identification of galangin and its metabolites in rat plasma

Chromatographic separations were achieved on a 4-μm Phenomenex Synergi Fusion-RP C18 column (2.0 mm i.d. × 50 mm) under 40°C with a pre-column 0.5-μm biocompatible inline filter. The mobile phase (delivered at 0.30 mL/min) was methanol/H_2_O containing 0.1‰ formic acid with a gradient program as follows: from 0% B to 2% B in 0.01 min, hold for 1 min; from 2% B to 35% B in 0.01 min, hold for 3 min; from 35% B to 90% B in 11 min; back to 2% B in 0.01 min; maintain 4.99 min [15].

The mass spectrometer was operated in the negative electrospray ionization (ESI) mode with selected multiple reaction monitoring (MRM) mode for all the analytes. The precursor-to-product ion pairs used for galangin, quercetin and kaempferol were *m/z* 268.9 → 108.0, 300.9 → 150.9 and 285.0 → 93.0, respectively, with a scan times of 20 ms for each ion pair. The phase II metabolites of galangin were also measured. The precursor-to-product ion pairs used for glucuronidated galangin (M_Gal-G), galangin diglucuronide (M_Gal-2G), galangin triglucuronide (M_Gal-3G), sulfated galangin glucuronide (M-Gal-G-S), sulfated galangin (M_Gal-S), galangin disulfate (M_Gal-2S), galangin trisulfate (M_Gal-3S), sulfated galangin diglucuronide (M-Gal-2G-S), disulfated galangin glucuronide (M-Gal-G-2S), methylated galangin (M-Gal-CH_3_), glucuronidated kaempferol (M_KMF-G), kaempferol diglucuronide (M_KMF-2G), sulfated kaempferol glucuronide (M_KMF-G-S), sulfated kaempferol (M_KMF-S), disulfated kaempferol (M_KMF-2S), glucuronidated quercetin (M_QCT-G), quercetin diglucuronide (M_QCT-2G), sulfated quercetin glucuronide (M_QCT-G-S), sulfated quercetin (M_QCT-S) and disulfated quercetin (M_QCT-2S) were m/z 444.9 → 268.9, 620.9 → 268.9, 796.9 → 268.9, 524.9 → 268.9, 348.9 → 268.9, 428.9 → 268.9, 508.9 → 268.9, 700.9 → 268.9, 604.9 → 268.9, 282.9 → 108.0, 461.0 → 285.0, 637.0 → 285.0, 541.0 → 285.0, 365.0 → 285.0, 445.0 → 285.0, 476.9 → 300.9, 652.9 → 300.9, 556.9 → 300.9, 380.9 → 300.9 and 460.9 → 300.9, respectively.

#### Quantitative analysis of free and total galangin in rat plasma

Chromatographic separations of prepared samples were achieved using a Phenomenex Kinetex 2.6 μm XB-C_18_ column (2.10 mm i.d × 50 mm) maintained at 40°C and coupled with a 0.5-μm biocompatible inline filter. The LC mobile phase composition and flow rate were the same as the above-mentioned. The gradient program was as follows: 0–0.3 min at 1% B; from 1% B to 100% B in 0.01 min (0.31 min) and maintained 2.7 min (0.31–3 min); from 100% B to 1% B in 0.01 min (3.01 min) and maintained 1 min (3.01–4 min). The mass spectrometer was operated in the negative ion ESI mode with MRM for galangin, M_Gal-G and IS. The precursor-to-product ion pairs used for galangin, M_Gal-G and chrysin were m/z 268.9 → 108.0, 444.9 → 268.9 and 253.0 → 142.9, respectively, with a scan time of 40 ms for each ion pair.

#### Assay validation

Assay validation was performed according to the US FDA guidance on bioanalytical method validation (http://www.fda.gov/downloads/Drugs/Guidances/ucm070107.pdf). The quality control samples were prepared from an independent weighing of the reference standard.

#### Data analysis

To determine the PK parameters, the concentration-time data were estimated by a non-compartmental method using the Kinetica 2000 software package (version 3.0; Innaphase Corp., Philadelphia, PA, USA). The *C*_max_ and *T*_max_ were observed values with no interpolation. The area under concentration-time curve up to the last measured time point (AUC_0→t_) was calculated by the trapezoidal rule method. The AUC_0→∞_ was generated by extrapolating the AUC_0→t_ to infinity. Results are expressed as the mean ± SD.

## Results and discussion

### Phase II conjugation metabolites of galangin in rat plasma

**Figure 2:**
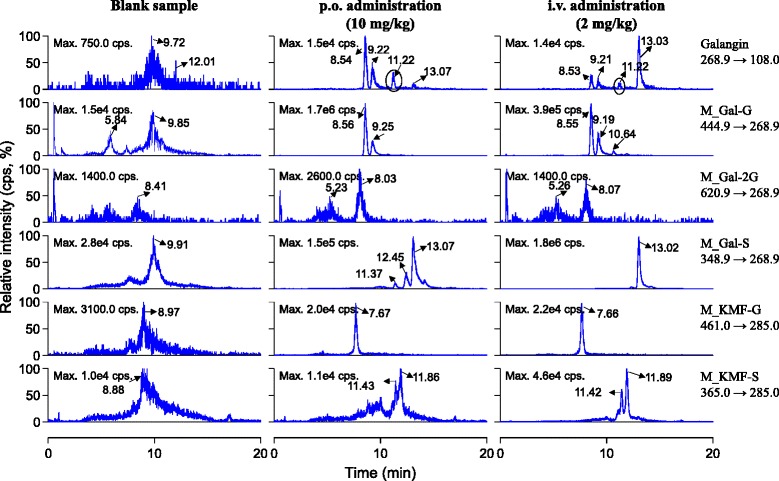
**Representative chromatograms for identification of glucuronidated and sulfated metabolites of galangin in rat plasma after a single p.o. dose (10 mg/kg) and an i.v. dose (2 mg/kg) of galangin solution.**

*In vitro* studies have revealed that galangin can be oxidized to kaempferol, which could be further transformed to quercetin by rat cytochrome P450 including CYP 1A1, CYP1A2 and CYP 2C9 [16,17], in human liver microsomes [18] and in human hepatocytes [11]. But, galangin mainly undergoes glucuronidation and sulfation in freshly plated human hepatocytes, with only trace amounts of oxidation. *In vivo* metabolism data of galangin is relatively scarce. In the current study, galangin and its oxidized products kaempferol and quercetin, as well as their glucuronidated or sulfated (mono-/di-/tri- type) metabolites in rat plasma after p.o. and i.v. dosing were monitored using LC-MS/MS with selected multiple reaction monitoring mode. As shown in Figure 2, the parent drug could be detected with retention time (Rt) about 11.22 min (upper panel). Two galangin glucuronides in the plasma samples occurred with Rt 8.54 min and 9.22 min, respectively. Moreover, the peak responses of the two metabolites were significantly higher than that of the parent drug. We could not exclude the occurrence of diglucuronide metabolites because two peaks (Rt 5.23 min and 8.03 min) with relatively weaker LC-MS/MS response were measured. After enzymatic hydrolysis by β-glucuronidase, these peaks disappeared; meanwhile, galangin was liberated from its conjugated forms. Sulfated galangin (Rt 13.02 min) was also measured. Interestingly, the peak responses of galangin glucuronides were significantly higher than those of sulfated galangin in plasma samples after p.o. dosing; however, the result was quite opposite after i.v. medication (Figure 2). In addition, methylated galangin was not detected. Galangin has three hydroxyl groups (3-, 5- and 7-) and these positions may be catalyzed by UDP-glucuronyltransferase and sulfatase theoretically. Thus, structures of the detected glucuronides and diglucuronide should be unambiguously identified in the future.

Our results revealed that the oxidized products of galangin (kaempferol and quercetin) were not measurable in the rat plasma samples (Figure 1). Also the glucuronides, diglucuronide, sulfated metabolites and sulfated diglucuronide metabolites of quercetin could not be detected. However, we found the occurrence of glucuronidated and sulfated quercetin in rat bile and urine samples after a single p.o. or i.v. administration of galangin solution to rats (data not shown). The phase II conjugation metabolites of kaempferol including glucuronide (Rt 7.66 min) and diglucuronide (Rt 11.42 and 11.89 min) could be detected (Figure 2). Overall, the major metabolites of galangin in rat plasma were glucuronides and sulfates; meanwhile, trace amounts of kaempferol phase II conjugates were also detected.

### Plasma pharmacokinetics of galangin

We have identified two galangin glucuronides in rat plasma; unfortunately there are no well-established standards for their quantification analysis. In this study, alternatively, free type and hydrolyzed type of galangin in rat plasma were detected using different plasma sample clean-up methods, *i.e.*, acetonitrile precipitation and with or without enzyme hydrolysis. In order to enhance analysis efficiency, a 4-min “pulse gradient” program was applied to analyze galangin and its glucuronidated metabolites. Because the run time was shortened from 20 min to 4 min, the two galangin glucuronides could not be separated; but formed a single peak (Rt 2.01 min) abutting the parent drug (Rt 2.10 min).

### Method validation results

**Figure 3:**
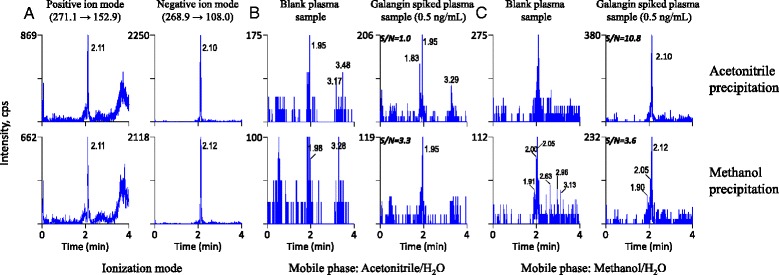
**Representative chromatograms for the optimum of LC-MS/MS conditions. (A)** Ionization mode selection, **(B)** mobile phase Acetonitrile/H_2_O and **(C)** mobile phase Methanol/H_2_O.

As shown in Figure 3, the ionization mode (Figure 3A), mobile phase composition (Figure 3B and C) and precipitation solvent (upper and bottom panel) were evaluated to achieve the highest LC-MS/MS response of galangin. Our results showed that the response in the negative ion mode was more sensitive (~3 times) than that of positive ion mode. With regard to mobile phase composition, methanol/H_2_O system was better than acetonitrile/H_2_O system. In addition, acetonitrile precipitation method resulted in higher sensitivity (signal-to-noisy ratio: S/N 10.8 *versus* 3.6) than methanol precipitation. To liberate galangin from its glucuronides, some rat plasma samples were treated with β-glucuronidase. Amounts of enzyme and the incubation time were assessed based on the reduction of galangin glucuronides present in rat plasma. Galangin glucuronides disappeared completely when the amounts of β-glucuronidase (2,000 unit/mL in pH 5 acetate buffers) were three times (volume-to-volume) of plasma samplesand incubated for 60 min.

The calibration curve (Y = 0.000177X + 1.47 × 10^-5^, weight coefficient 1/×^2^) was linear over the measured range of 2-2000 ng/mL for free type of galangin with correlation coefficient of 0.994. The LLOQ, precision and accuracy was 2 ng/mL, 9.18% and 101%, respectively. Similar results were obtained for the quantification of galangin in samples after the treatment of β-glucuronidase hydrolysis. The LLOQ was 2 ng/mL, with a precision of 11.4% and accuracy of 102% for this compound.

Intra- and inter-run precision and accuracy data are shown in Table 1. Accuracy ranged from 96.4% to 107%, which was up to the FDA standard. Intra- and inter-batch deviations ranged from 3.07% to 8.14% and from 6.00% to 8.87%, respectively. Therefore, the precision and accuracy data were within the acceptable criteria and allowed the accurate quantification of the galangin in rat plasma.

**Table 1:** **Precision and accuracy of the galangin in rat plasma (n = 5)**

**Analyte**	**Spiked concentration (ng/mL)**	**Intra-day**			**Inter-day**		
		**Measured (ng/mL)**	**RSD (%)**	**Accuracy (%)**	**Measured (ng/mL)**	**RSD (%)**	**Accuracy (%)**
Galangin	15	14.6 ± 8.19	8.14	97.3 (8.07)	14.5 ± 1.29	8.87	96.4 (8.76)
	600	625 ± 40.0	6.40	104 (6.42)	610 ± 36.6	6.00	102 (6.04)
	1200	1292 ± 39.6	3.07	107 (3.33)	1219 ± 87.0	7.14	102 (7.08)

The extraction recoveries (Table 2) were in the range of 34.8-51.6% for galangin and 31.8% for IS indicating that the enzymatic hydrolysis method could offer good extraction efficiency for these analytes in rat plasma matrices. Matrix effects and extraction efficiencies were assessed in quintuplicate by comparing analyte peak areas of across three different sample sets [19]. Matrix effects of galangin and IS were found to be acceptable in various plasma matrices from different sources (86.6–90.2%, Table 2).

**Table 2:** **Matrix effect and extraction recovery of galangin and IS in rat plasma (n = 5)**

**Analyte**	**Peak area (× 10** ^**3**^ **)**									
	**Set 1**		**Set 2**		**Set 3**		**Matrix effect**		**Extraction efficiency**	
	**Mean ± SD**	**RSD (%)**	**Mean ± SD**	**RSD**	**Mean ± SD**	**RSD (%)**	**Mean (%)**	**RSD (%)**	**Mean (%)**	**RSD (%)**
Galangin (ng/mL)										
15	3.44 ± 0.39	11.5	2.98 ± 0.08	2.87	1.04 ± 0.11	10.4	86.6	2.87	34.8	10.4
600	97.0 ± 5.14	5.30	86.5 ± 4.48	5.19	37.8 ± 1.62	4.28	89.1	5.19	43.7	4.28
1200	168 ± 16.0	9.53	147 ± 5.37	3.64	76.0 ± 3.14	4.13	87.6	3.64	51.6	4.13
IS (ng/mL)										
500	279 ± 35.4	12.7	251 ± 9.52	3.79	80.0 ± 5.24	6.55	90.2	3.79	31.8	6.55

As shown in Table 3, the storage of plasma samples at room temperature for 4 h (pretreatment) did not alter signal responses of galangin. Also, processed samples (post-treatment) were stable at auto-sampler room for 12 h. The accuracy between initial and final analysis ranged from 86.0% to 96.5%, with RSD between 4.72% and 11.5%. Moreover, three free-thaw cycles did not influence the stability of galangin.

**Table 3:** **Stability of the galangin in rat plasma (n = 5)**

**Spiked concentration (ng/mL)**	**Short-term stability (4 h at room temperature)**		**Autosampler stability (12 h at room temperature)**		**Freeze-thaw stability (3 cycles)**	
	**Mean ± SD**	**RSD (%)**	**Mean ± SD**	**RSD (%)**	**Mean ± SD**	**RSD (%)**
15	15.1 ± 1.9 (12.9)	100 (12.8)	14.5 ± 1.70 (11.7)	96.5 (11.5)	15.1 ± 1.5 (10.2)	101 (10.3)
600	535 ± 43.2 (8.08)	89.1 (7.92)	592 ± 33.2 (5.61)	98.7 (5.62)	527 ± 13.9 (2.63)	87.9 (2.64)
1200	1070 ± 18.9 (1.76)	89.3 (1.84)	1150 ± 52.3 (4.65)	86.0 (4.72)	1086 ± 23.2 (2.14)	90.6 (2.20)

### Systemic exposure to and pharmacokinetics of galangin

**Figure 4:**
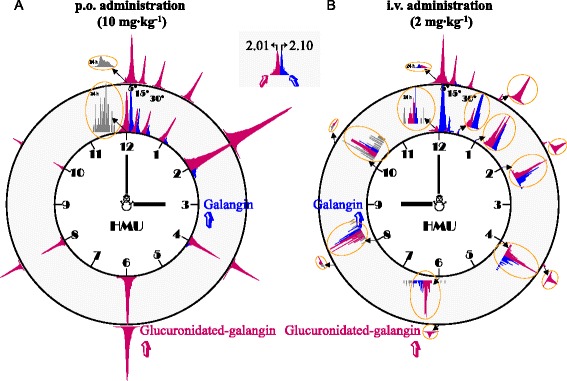
**Representative peak responses of galangin (peaks filled in blue) and its glucuronides (peaks filled in rose) over time after a p.o. dose (10 mg/kg; A) or an i.v. dose of galangin (2 mg/kg; B) in rats.**

**Figure 5:**
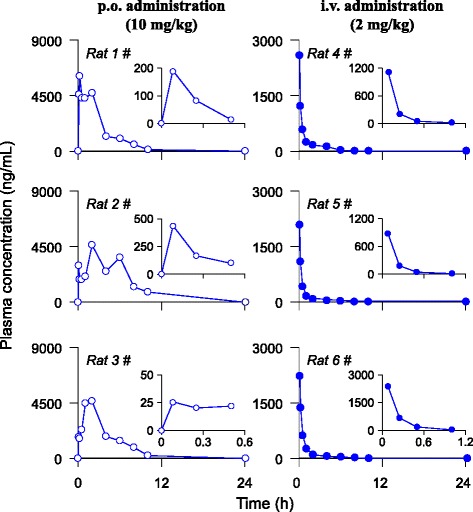
**Plasma concentration-time profiles of total type of galangin after a p.o. dose (10 mg/kg) or an i.v. dose of galangin (2 mg/kg) in rats.** The right and smaller inserts show the plasma concentration-time profiles of free type of galangin after the same dosing.

Figure 4 depicts the peak responses of galangin (peaks filled in blue) and its glucuronides (peaks filled in rose) over time after a p.o. dose (Figure 4A) or an i.v. dose of galangin (Figure 4B) to rats. 5 min after i.v. dosing, the peak response of galangin (Rt 2.10 min) was predominant; meanwhile the peak of galangin glucuronide (Rt 2.01 min) was minor. Subsequently, peak responses of both the galangin and its glucuronides declined rapidly. The peak height of parent drug and its glucuronidated metabolites were almost equal at 1 h after dosing. At 2 h after medication the parent drug was not detectable. In addition, the peak response of galangin glucuronide reached its maximum level at 5 min after i.v. administration and then decreased rapidly. After oral administration, galangin was absorbed quickly from gastrointestinal tract and peaked at 5 min; meanwhile, the glucuronides were also detected with comparable peak responses with parent drug. Whereafter, the parent drug decayed rapidly but the glucuronides could be monitored in the rat plasma samples up to 24 h after dosing. The corresponding plasma concentration-time profiles of the free type of galangin in rats following a single p.o. dose (rat 1#, 2# and 3#) or a single i.v. bolus (rat 4#, 5# and 6#) of the galangin solution are shown in right and smaller inserts of Figure 5.

After enzymatic hydrolysis, the aglycone was liberated from its conjugated forms. The plasma concentration-time profiles of hydrolyzed type of galangin after i.v. and p.o. dosing in rats are shown in Figure 5. The key PK parameters for both type of galangin are summarized in Table 4. The maximal concentrations of both type of galangin were comparable after i.v. dosing indicating the parent drug molecules were predominant at that time. This was not true for oral administration route, where the mean *C*_max_ value of total type of galangin was 23 times greater than that of free drug. Therefore, the glucuronidated galangin was the main principle circulating in the bloodstream after oral medication. Correspondingly, the mean oral bioavailability of free type of galangin was about only 3.67%; on the contrast, the value for hydrolyzed type of galangin reached 380%. The utilized β-glucuronidase solution contains β-glucuronidase (≥100,000 U∙mL^-1^) and sulfatase (7,500 U∙mL^-1^) resulting in incomplete hydrolysis of galangin sulfates present in rat plasma samples after i.v. treatment, which, at least in part, contributed to the higher oral bioavailability of hydrolyzed type of galangin. In addition, the multiple peaking phenomena present in the plasma concentration-time curves of p.o. medication route, rather than i.v. treatment, likely resulted from physiological factors, including – but not limited to – site-specific intestinal absorption, instead of enterohepatic circulation, of the galangin [20]. Because galangin glucuronides and sulfates were mainly eliminated via renal excretion, their enterohepatic circulation might be neglectable.

**Table 4:** **Pharmacokinetic data of galangin after administration to rats (n = 3)**

**PK parameters**	**i.v. administration**	**p.o. administration**
	**(2 mg/kg)**	**(10 mg/kg)**
***Free type of galangin***		
*C*_max_ or *C*_5min_ (ng/mL)	1456 + 802	219 ± 207
*T*_max_ (h)	0.083	0.083
AUC_0-t_ (h•ng/mL)	265 ± 176	48.7 ± 44.1
AUC_0-∞_ (h ng/mL^-1^)	271 ± 179	80.8 ± 40.5
*t*_1/2_ (h)	0.21 ± 0.02	-
MRT (h)	0.18 ± 0.01	1.28 ± 1.75
CL_tot, p_ (L•h/kg)	6.86 ± 3.32	-
*V*_ss_ (L/kg)	1.20 ± 0.55	-
*F* (%)	-	3.67 ± 3.33
***Hydrolyzed type of galangin after enzyme hydrolysis treatment***		
*C*_max_ or *C*_5min_ (ng/mL)	2273 + 265	5103 ± 829
*T*_max_ (h)	0.083	0.083, 2
AUC_0-t_ (h ng/mL)	1169 ± 360	22184 ± 4482
AUC_0-∞_ (h ng/mL^-1^)	1188 ± 339	22214 ± 4482
*t*_1/2_ (h)	1.37 ± 0.69	-
MRT (h)	1.19 ± 0.51	4.03 ± 1.09
CL_tot, p_ (L•h/kg)	1.59 ± 0.47	-
*V*_ss_ (L/kg)	1.73 ± 0.42	-
*F* (%)	-	380 ± 77

The routes of administration demonstrated significant influences on the systemic exposure to the galangin and its metabolites. Given that the positive pharmacological activities confirmed via *in vitro* and *in vivo* studies, it remains open to question what exactly makes them run. The premise is that enough free parent drug molecules could reach the certain target when galangin works as active principle. Under this situation, there should be a dynamic equilibrium of galangin between systemic circulation and certain targets. Conjugated galangin circulates in the bloodstream and transforms into aglycone before arriving at the targets. Mukai *et al.* found that quercetin glucuronide could be deconjugated to quercetin by microglial MG-6 cells [21]. As for galangin, further studies on tissue and cellular distribution should be done in the future in order to test the hypothesis. On the other hand, the galangin glucuronides and sulfates could work as active principle and play a critical role in the claimed activities. Some studies have confirmed that the quercetin glucuronides have various pharmacological roles, including anti-inflammatory effects [22,23], anti-atherosclerotic roles [24-26], immune-modulatory activity [27], antioxidant effects [28,29] and anti-proliferative effect [30]. Therefore, biological activities of galangin conjugate (chemically synthesized or separated from biosamples) need to be evaluated.

## Conclusions

In summary, this study provided direct evidence that the routes of administration show significant influences on the systemic exposure level of galangin and its metabolites. Galangin was preferentially glucuronidated after p.o. dosing but sulfated after i.v. medication. Kaempferol conjugates were measured indicating that oxidation reaction occurred; however, both glucuronidation and sulfation of galangin were more efficient. LC-MS/MS methods were developed and validated for quantification of galangin both in its unmetabolized form and hydrolyzed form liberated from its glucuronides via β-glucuronidase hydrolysis, respectively. The oral bioavailability of parent galangin was very low. Further research is needed to unambiguously identify the structures of galangin conjugates and to evaluate the biological roles of these metabolites.
